# Precision-guided treatment in high-risk pediatric cancers

**DOI:** 10.1038/s41591-024-03044-0

**Published:** 2024-06-06

**Authors:** Loretta M. S. Lau, Dong-Anh Khuong-Quang, Chelsea Mayoh, Marie Wong, Paulette Barahona, Pamela Ajuyah, Akanksha Senapati, Sumanth Nagabushan, Alexandra Sherstyuk, Ann-Kristin Altekoester, Noemi A. Fuentes-Bolanos, Veronica Yeung, Ashleigh Sullivan, Natacha Omer, Yonatan Diamond, Sophie Jessop, Lauren Battaglia, Nataliya Zhukova, Louise Cui, Angela Lin, Andrew J. Gifford, Emmy D. G. Fleuren, Luciano Dalla-Pozza, Andrew S. Moore, Seong-Lin Khaw, David D. Eisenstat, Nicholas G. Gottardo, Paul J. Wood, Heather Tapp, Frank Alvaro, Geoffrey McCowage, Wayne Nicholls, Jordan R. Hansford, Neevika Manoharan, Rishi S. Kotecha, Marion K. Mateos, Richard B. Lock, Vanessa Tyrrell, Michelle Haber, Toby N. Trahair, Mark J. Cowley, Paul G. Ekert, Glenn M. Marshall, David S. Ziegler

**Affiliations:** 1 Children’s Cancer Institute, Lowy Cancer Research Centre, UNSW Sydney, Sydney, New South Wales Australia; 2 School of Clinical Medicine, UNSW Medicine & Health, UNSW Sydney, Sydney, New South Wales Australia; 3 Kids Cancer Centre, Sydney Children’s Hospital, Sydney, New South Wales Australia; 4 Children’s Cancer Centre, Royal Children’s Hospital, Melbourne, Victoria Australia; 5 Department of Paediatrics, Murdoch Children’s Research Institute, University of Melbourne, Melbourne, Victoria Australia; 6 Oncology Services Group, Children’s Health Queensland Hospital & Health Service, Brisbane, Queensland Australia; 7 Frazer Institute, Faculty of Medicine, The University of Queensland, Brisbane, Queensland Australia; 8 Michael Rice Centre for Haematology and Oncology, Women’s and Children’s Hospital, Adelaide, South Australia Australia; 9 Children’s Cancer Centre, Monash Children’s Hospital, Melbourne, Victoria Australia; 10 Centre for Cancer Research, Hudson Institute of Medical Research, Melbourne, Victoria Australia; 11 Department of Paediatrics, School of Clinical Sciences at Monash Health, Faculty of Medicine, Nursing and Health Sciences, Monash University, Melbourne, Victoria Australia; 12 Anatomical Pathology, NSW Health Pathology, Prince of Wales Hospital, Sydney, New South Wales Australia; 13 Cancer Centre for Children, The Children’s Hospital at Westmead, Sydney, New South Wales Australia; 14 Department of Paediatric and Adolescent Oncology and Haematology, Perth Children’s Hospital, Perth, Western Australia Australia; 15 Telethon Kids Cancer Centre, Telethon Kids Institute, University of Western Australia, Perth, Western Australia Australia; 16 Children’s Cancer and Blood Disorders, John Hunter Children’s Hospital, University of Newcastle, Newcastle, New South Wales Australia; 17 Department of Paediatrics, Faculty of Medicine, Dentistry and Health Sciences, University of Melbourne, Melbourne, Victoria Australia; 18 South Australia Health and Medical Research Institute, Adelaide, South Australia Australia; 19 South Australian immunoGENomics Cancer Institute, University of Adelaide, Adelaide, South Australia Australia; 20 Curtin Medical School, Curtin University, Perth, Western Australia Australia; 21 UNSW Centre for Childhood Cancer Research, UNSW Sydney, Sydney, New South Wales Australia; 22 Cancer Immunology Program, Peter MacCallum Cancer Centre, Melbourne, Victoria Australia

**Keywords:** Translational research, Cancer genomics, Paediatric cancer, Targeted therapies

## Abstract

Recent research showed that precision medicine can identify new treatment strategies for patients with childhood cancers. However, it is unclear which patients will benefit most from precision-guided treatment (PGT). Here we report consecutive data from 384 patients with high-risk pediatric cancer (with an expected cure rate of less than 30%) who had at least 18 months of follow-up on the ZERO Childhood Cancer Precision Medicine Program PRecISion Medicine for Children with Cancer (PRISM) trial. A total of 256 (67%) patients received PGT recommendations and 110 (29%) received a recommended treatment. PGT resulted in a 36% objective response rate and improved 2-year progression-free survival compared with standard of care (26% versus 12%; *P* = 0.049) or targeted agents not guided by molecular findings (26% versus 5.2%; *P* = 0.003). PGT based on tier 1 evidence, PGT targeting fusions or commenced before disease progression had the greatest clinical benefit. Our data show that PGT informed by comprehensive molecular profiling significantly improves outcomes for children with high-risk cancers. ClinicalTrials.gov registration: NCT03336931

## Abstract (web summary)

In the observational ZERO Childhood Cancer Precision Medicine Program PRecISion Medicine for Children with Cancer (PRISM) trial, children with high-risk cancer were treated with molecular tumor board-recommended therapies, resulting in overall clinical response rates that translated into survival benefit after long-term follow-up.

## Main

The development of next-generation sequencing (NGS) in conjunction with targeted anticancer therapies has allowed the delivery of precision medicine, selecting for the molecular drivers of a patient’s cancer. Pediatric precision oncology studies have identified potentially targetable molecular findings in over 65% of children with high-risk cancers^1–5^. However, clinical uptake of matched targeted therapies in these studies was generally low, ranging from 10% to 33%. One reason for low clinical uptake is physician uncertainty regarding the efficacy and benefit–risk balance of precision-guided treatment (PGT).

It is unclear which children with high-risk cancers are most likely to benefit from PGT and whether PGT improves survival. Early studies described the potential for the clinical benefit of PGT, but without objective response (OR) assessments or long follow-up^6–9^. More recently, the INFORM study showed improved survival outcomes limited to patients with high-evidence targets^2^. The GAIN study suggested that responses may be restricted to treatments targeting activating fusions^3^. Similarly, the MAPPYACTS study showed that treatments directed at higher-tier evidence led to improved response rates but did not report survival outcomes^4^. Thus, to our knowledge, no studies have evaluated both OR and survival outcomes, and there are limited data to determine which patients should receive PGT and when.

The ZERO Childhood Cancer Precision Medicine Program PRecISion Medicine for Children with Cancer (PRISM) trial used whole-genome sequencing (WGS) (paired tumor-germline), and transcriptomic sequencing and DNA methylation, to identify molecular targets in high-risk cancers. Therapeutic options for potentially actionable aberrations for each patient were discussed in a national molecular tumor board (MTB). In this article, we report a comprehensive outcome analysis including both response and survival for the first 384 high-risk patients with at least 18 months of follow-up and identify prognostic factors to help determine the most effective PGT strategies.

## Results

### Patients and baseline characteristics

**Fig. 1:**
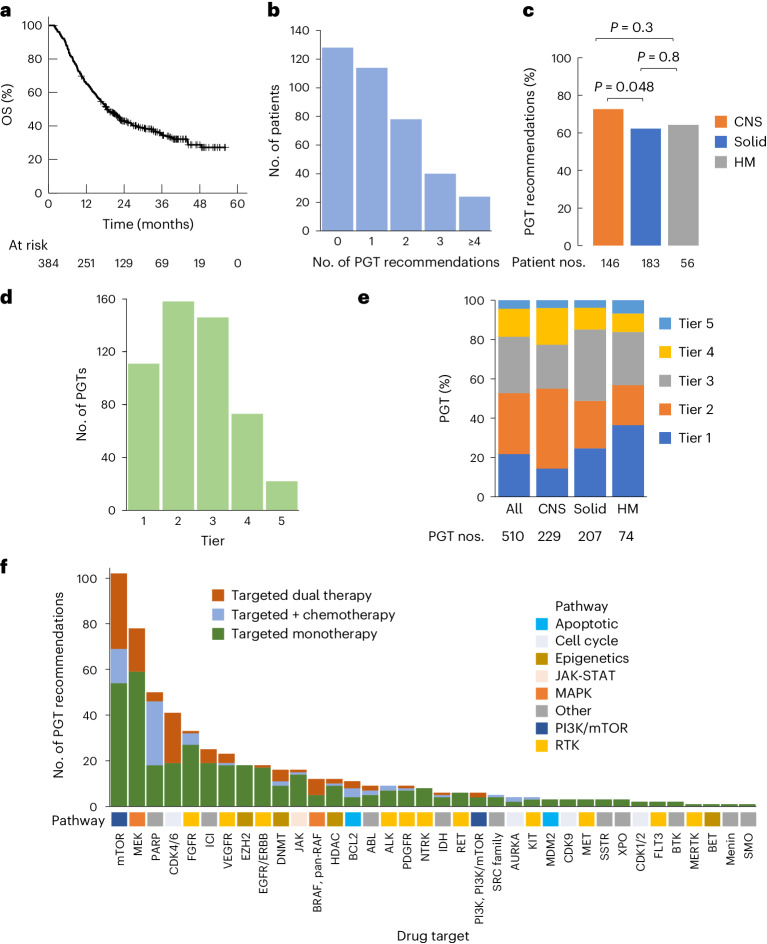
Clinical uptake of PGT. **a**, OS of 384 patients with high-risk cancers. **b**, Number of PGT recommendations per patient. **c**, Frequency of PGT recommendations according to cancer type. **d**, Number of PGT recommendations from the highest level of supporting evidence to the lowest (tier 1, clinical evidence in the same cancer; tier 2, clinical evidence in a different cancer; tier 3, preclinical evidence in the same cancer; tier 4, preclinical evidence in a different cancer; tier 5, consensus opinion). **e**, Distribution of PGT recommendation tier according to cancer type. **f**, Types of targeted therapy in relation to the drug target. Targeted therapy was categorized into targeted monotherapy, targeted dual therapy and targeted agent in combination with chemotherapy. The corresponding molecular pathway for each of the drug targets is shown. **c**, *P* values for comparison of proportions using a two-sided chi-squared test.

**Extended Data Fig. 1:**
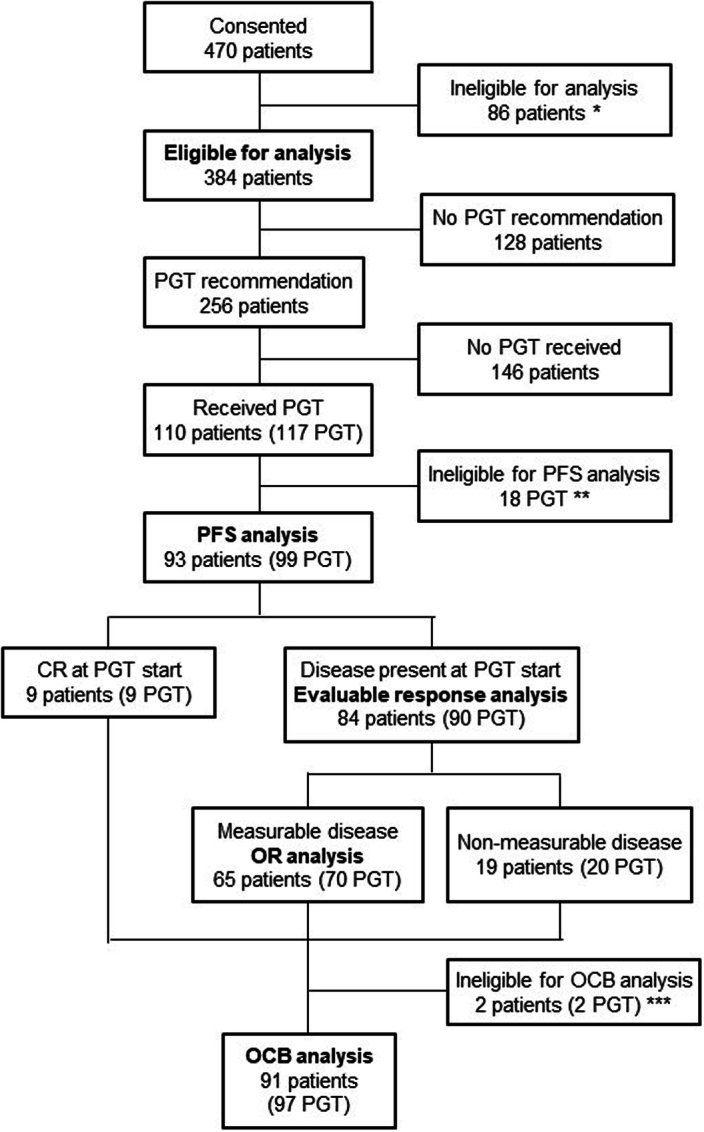
CONSORT diagram. Consort diagram of 470 patients consented in the PRISM study between Sep 2017 and Dec 2020. 384 patients were eligible for outcome analysis. * ineligible due to non-high-risk cancer diagnosis, lack of appropriate sample or death prior to presentation at the molecular tumour board. ** ineligible due to treatment duration <4 weeks, disease progression within the first 4 weeks of treatment or no response evaluation. *** not evaluable for OCB due to cessation of treatment before 24 weeks in the absence of disease progression where stable disease was best response. CR, complete remission; OCB, objective clinical benefit; OR, objective response of measurable disease; PGT, precision-guided treatment; PFS, progression-free survival.

Four hundred and seventy consecutively enrolled patients with high-risk cancers (expected cure rate lower than 30% assessed by both referring oncologist and central review) were consented for the PRISM study between 14 September 2017 and 31 December 2020. Eighty-six patients were ineligible because of a non-high-risk cancer diagnosis on central review, lack of appropriate sample or death before MTB presentation (Extended Data Fig. 1). Hence, 384 patients discussed at the MTB were included in this analysis. The molecular profile of 181 of these patients has been described previously^1^. At the time of data cutoff on 30 June 2022, 244 patients were deceased, two were lost to follow-up and the remaining 138 had at least an 18-month follow-up from enrollment (median = 33.7; range: 18.2–56.9 months). The 3-year overall survival (OS) of the 384-patient cohort was 34% (95% confidence interval (CI) = 29–40%) (Fig. 1a).

Of the 384 eligible patients, 160 patients were enrolled at first cancer diagnosis, 184 patients at first relapse and 40 patients after two or more previous relapses (Extended Data Table 1 and Supplementary Data 1). The cohort consisted of 146 central nervous system (CNS) tumors, 183 solid tumors and 56 hematologic malignancies (HMs). One patient with a germline mutation in the *TP53* gene had two synchronous tumors analyzed (medulloblastoma (MB) and osteosarcoma (OST)). Median age at enrollment was 10.9 years (range 0.1–46 years), including 14 adults (aged older than 21 years) with pediatric-type cancers.

All patients had at least one somatic NGS assay performed. Both WGS and whole-transcriptome sequencing (WTS) were successfully conducted on 319 of 385 samples (83%). WGS alone was performed on 54 samples, targeted panel on ten and targeted panel plus WTS on two cases either because of insufficient DNA or RNA or because only formalin-fixed paraffin-embedded tissue was available (Extended Data Table 1). DNA methylation profiling was performed in 298 of 329 CNS tumors or sarcomas. Germline WGS was performed on 374 patients and germline targeted panel on ten patients.

### Identification of therapeutic targets

Molecular findings were classified as reportable or actionable as described previously^1^ and discussed in the national MTB. A five-tier system was used to assign the strength of the PGT recommendation (Methods and Supplementary Data 2). PGT was recommended only if age-specific drug safety data were available and there was a possibility of drug access in Australia via registered indication, clinical trials, compassionate access or off-label use. Two hundred and fifty-six patients (67%) received at least one PGT recommendation, with a total of 510 PGT recommendations made (Fig. 1b). The recommendation rate was significantly higher for CNS tumors than solid tumors (73% versus 62%; *P* = 0.048) (Fig. 1c). While 53% of the recommendations had supporting clinical evidence (tiers 1 and 2), 43% were derived from preclinical evidence (tiers 3 and 4) (Fig. 1d). CNS tumors had significantly fewer tier 1 recommendations compared with solid tumors (14% versus 25%; *P* = 0.007) and HMs (14% versus 36%; *P* < 0.0001) (Fig. 1e). The 510 PGT recommendations consisted of 74% targeted monotherapy, 12% targeted dual therapy, 13% targeted and chemotherapy combination, and 1% chemotherapy alone. Therapies targeting the phosphoinositide 3-kinase (PI3K)/mammalian target of rapamycin (mTOR) (20%) and mitogen-activated protein kinase (MAPK) (15%) pathways were most frequently recommended, followed by poly(ADP-ribose) polymerase (PARP) (10%) and cyclin-dependent kinase 4 (CDK4) and CDK6 inhibitors (8%) (Fig. 1f). Of the receptor tyrosine kinases (RTKs), fibroblast growth factor receptor (FGFR) (28%) was the most common target followed by vascular endothelial growth factor (VEGF)/vascular endothelial growth factor receptor (VEGFR) (20%) and epidermal growth factor receptor (EGFR)/ERBB (16%).

### Clinical uptake and drug access for PGT

Of the 256 patients with a PGT recommendation, 110 (43%) were subsequently treated with a PGT, with a median time of returning results at 6.6 weeks. Seventy percent of PGTs were commenced within 3 months of the MTB, with a median start time of 9 weeks (1 day–2.5 years). Three patients started treatment before MTB after rapid communication of results to the treating clinician. In total, 117 PGTs were administered to 110 patients, with six patients receiving two or more consecutive PGTs. The early clinical responses to 37 of these PGTs were reported previously^1^. Of note, clinical testing for the specific PGT target was only available for 13 targets and was performed in ten. For these ten patients, only six returned a positive test, with four returning a negative or equivocal result. Thus, for 95% of patients receiving PGT, their driver either could not be or was not detected through locally available testing (Supplementary Table 1).

The mechanism according to which patients gained access to the 117 PGTs included compassionate access in 42 (36%), funding via the local clinical institution in 39 (33%), clinical trial enrollment in 19 (16%), funding from the government Pharmaceutical Benefit Scheme in ten (9%), cost sharing arrangement between hospital and drug company in five (4%) and self-funded in two (2%) (Supplementary Data 3).

### Clinical benefit of PGT

**Fig. 2:**
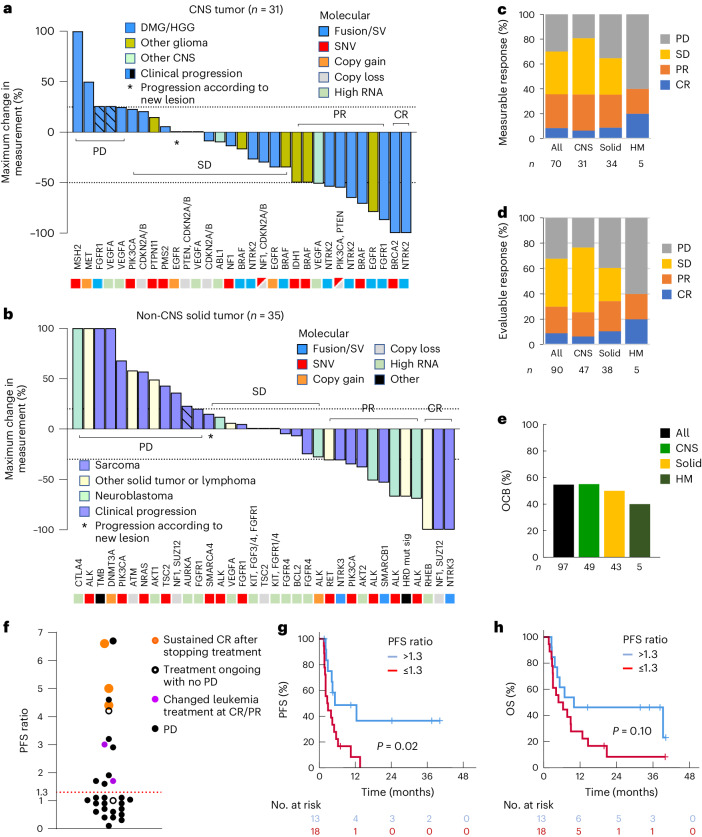
Patients receiving PGT experienced a clinical response. **a**, Waterfall plot for 31 CNS tumors with measurable disease at the start of a PGT. Treatment response was evaluated using the RANO criteria. The dotted lines at 25% and −50% delineate the category of response (≥25%, PD; 25 to −50%, SD; −50% or lower to −99%, PR; −100%, CR). **b**, Waterfall plot for 35 solid tumors with measurable disease at the start of a PGT. Treatment response was evaluated using the RECIST or PERCIST criteria. The dotted lines at 25% and −30% delineate the category of response (≥25%, PD; 25 to −30%, SD; −30% or lower to −99%, PR; −100%, CR). **c**, Response according to cancer type to 70 PGTs given with measurable disease. **d**, Response according to cancer type to 90 PGTs with evaluable disease (70 measurable and 20 non-measurable). **e**, OCB rate in 97 PGTs. OCB was defined as CR, PR and SD of 24 weeks’ duration or longer. **f**, PFS ratio for 31 PGTs. The PFS ratio was defined as the PFS duration of the PGT to that of a previous treatment in the same patient. A PFS ratio greater than 1.3 (above the dotted line) represents prolongation of the progression-free period for more than 30% by the PGT compared to a previous treatment. The color of the dots denotes the clinical course. **g**,**h**, PFS (**g**) and OS (**h**) stratified according to PFS ratio. A two-sided log-rank test was used to compare the Kaplan–Meier survival curves.

Of the 117 administered PGTs, 99 (received by 93 patients) were eligible for outcome analysis (Supplementary Data 3). Eighteen PGTs were excluded from the analysis, including 14 patients whose treatment duration was less than 4 weeks (Extended Data Fig. 1). Disease responses were evaluated using the Response Evaluation Criteria in Solid Tumors (RECIST), Response Assessment in Neuro-Oncology (RANO) or Positron Emission Tomography Response Criteria in Solid Tumors (PERCIST) criteria. Measurable disease was present at the start of 70 PGTs, with complete responses (CRs) observed in six (9%), partial responses (PRs) in 19 (27%), stable disease (SD) in 24 (34%) and progressive disease (PD) in 21 (30%) (Fig. 2a–c). The OR rate (ORR) (CR or PR) was similar for CNS and solid tumors (35% versus 34%). In addition, 20 PGTs (19 patients) were commenced for evaluable but non-measurable disease, with two CRs, ten SDs and eight PDs. Thus, the outcome for 90 evaluable PGTs (70 measurable and 20 non-measurable, excluding nine with no evidence of disease at the start of PGT) was CR in 9%, PR in 21%, SD in 38% and PD in 32% (Fig. 2d).

The duration of disease control can be meaningful for patients with high-risk cancers receiving new therapies; objective clinical benefit (OCB) (CR, PR and sustained SD for 24 weeks or longer) has been used as an endpoint in clinical trials of targeted agents^10,11^. Therefore, we evaluated OCB for 97 PGTs, including nine PGTs commenced with no evidence of disease (Extended Data Fig. 1). OCB was observed in 55% (53 of 97) of PGTs and was similar across tumor types (Fig. 2e).

The intra-patient progression-free survival (PFS) ratio has been used to compare the efficacy of PGT with previous treatments for the same patient, with clinical benefit defined as a PFS ratio greater than 1.3 (refs. ^12,13^). Thirty-one patients treated with PGT were assessable for PFS ratio and 42% (95% CI = 25–61%) had a PFS ratio greater than 1.3 (Fig. 2f). To determine whether a prolonged PFS ratio correlated with improved survival, we compared patients with a PFS ratio greater than 1.3 with those with a ratio of 1.3 or lower and found that they had a significantly improved PFS (2-year PFS 36% versus 0%; *P* = 0.02) (Fig. 2g). There was a similar difference in OS that did not reach statistical significance (2-year OS 46% versus 8.3%; *P* = 0.10) (Fig. 2h).

### PGT improved outcomes compared to other treatments

**Fig. 3:**
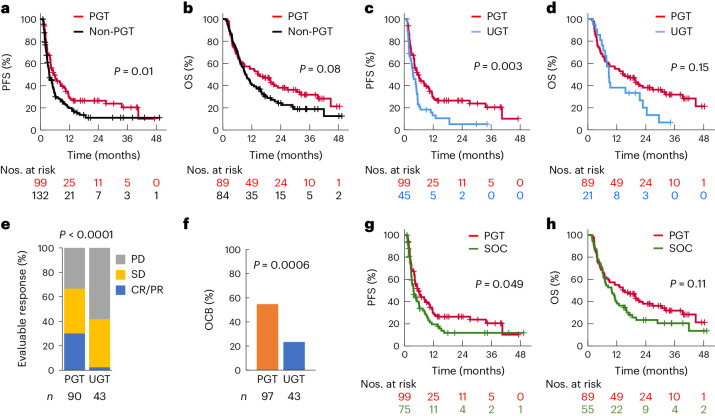
Superior clinical outcome of PGT. **a**,**b**, PFS (**a**) and OS (**b**) stratified according to PGT and non-PGT commenced at any point after MTB discussion. **c**,**d**, PFS (**c**) and OS (**d**) stratified according to PGT and UGT, that is, new therapy not molecularly guided. **e**, Response to PGT and UGT in patients with evaluable disease. **f**, OCB rate in PGT and UGT. OCB was defined as CR, PR and SD of 24 weeks’ duration or longer, and ongoing CR of 24 weeks or longer for patients who were in CR at the start of treatment. A two-sided chi-squared test was used to compare the CR and PR rate in **e** and OCB rate in **f**. **g**,**h**, PFS (**g**) and OS (**h**) stratified according to PGT and SOC. For OS comparison, a patient was categorized according to the first treatment that was initiated after MTB discussion. The Kaplan–Meier survival curves were compared using a two-sided log-rank test.

To understand whether PGT improved outcomes compared with other therapies, we next compared the outcomes for patients who received PGT versus non-PGT, that is, other therapies not recommended by the MTB, including standard of care (SOC) treatment and new or targeted therapies not guided by molecular findings and not recommended by the MTB, termed unguided therapy (UGT) in this study. One hundred and seventy-three patients whose treatment commenced after MTB and were evaluable for disease progression (treatment duration 4 weeks or longer and progression-free for 4 weeks or longer) were included in the survival analysis. Eighty-nine and 84 patients received a PGT or non-PGT as first treatment after MTB, respectively, and were compared for OS. For the PFS analysis, 99 PGTs were compared with 132 non-PGTs (75 SOC, 45 UGT and 12 other experimental treatments). Treatment with PGT resulted in significantly improved PFS when compared with non-PGT (2-year PFS 27% versus 11%; *P* = 0.01) (Fig. 3a), whereas the difference in OS did not achieve statistical significance (2-year OS 38% versus 24%; *P* = 0.08) (Fig. 3b), perhaps because of different salvage therapies (Supplementary Table 2).

**Extended Data Fig. 2:**
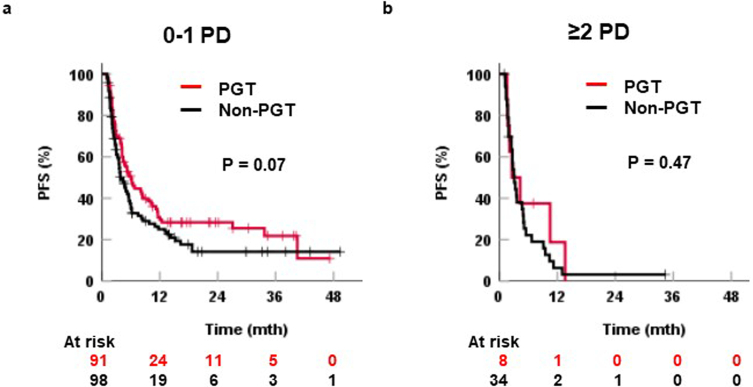
Progression-free survival of precision-guided treatment (PGT) and non-PGT by disease status. Progression-free survival (PFS) of PGT and non-PGT for (**a**) treatment given at no disease progression (PD) or one PD only since study enrolment and (**b**) treatment received at ≥2 PD since study enrolment. Log rank test is used to compare the Kaplan Meier survival curves.

We asked whether disease status could impact treatment. Of 99 PGTs, 49 were given before disease progression since study enrollment, 42 after one disease progression and eight after two or more episodes. Of 132 non-PGTs, 22 were given before disease progression since study enrollment, 76 after one disease progression and 34 after two or more episodes. For treatments given after no or one episode of progression, the 2-year PFS was 28% for PGT and 14% for non-PGT (*P* = 0.07). For treatments received after two or more disease progressions, there was no difference in PFS (2-year PFS 0% versus 3%; *P* = 0.47) (Extended Data Fig. 2).

It is possible that PGT was superior to the other therapies as these patients received new agents rather than standard cytotoxic therapies. Therefore, we compared the outcomes for PGT with UGT. Instances of patients receiving UGT included those enrolled on phase I trials of agents not requiring biomarkers, or treatments based on previous clinical trial data, for example, pazopanib for sarcoma and venetoclax for leukemia. A total of 45 UGTs were commenced after MTB in 36 patients (Extended Data Table 2 and Supplementary Data 4). Two UGTs were excluded from the response evaluation because the patients were disease-free at the start of treatment. PGT resulted in significantly improved PFS compared with UGT (2-year PFS 26% versus 5.2%; *P* = 0.003) (Fig. 3c), whereas the difference in OS did not achieve statistical significance (2-year OS 38% versus 20%; *P* = 0.15) (Fig. 3d). The response rate (CR/PR) was significantly higher after PGT compared with UGT (30% versus 2.3%; *P* < 0.0001) (Fig. 3e). Similarly, a higher OCB rate was observed with PGT versus UGT (55% versus 23%; *P* = 0.0003) (Fig. 3f).

SOC treatment was commenced after MTB discussion in 65 patients. SOC was defined as treatment routinely used in a tumor type or treatment reported to have proven clinical activity, for example, irinotecan (IRN) or temozolomide (TMZ) for relapsed Ewing’s sarcoma (EWS) and FLAG-Ida (fludarabine, cytarabine, idarubicin, granulocyte-colony stimulating factor) for relapsed acute leukemia. Fifty-five of these patients received SOC as first treatment after MTB and were included in the SOC group for OS comparison. A total of 75 SOC regimens were evaluated for PFS. We found that PGT led to significantly improved PFS compared to SOC (2-year PFS 26% versus 12%; *P* = 0.049) (Fig. 3g), whereas the difference in OS did not achieve significance (2-year OS 38% versus 23%; *P* = 0.11) (Fig. 3h).

**Extended Data Fig. 3:**
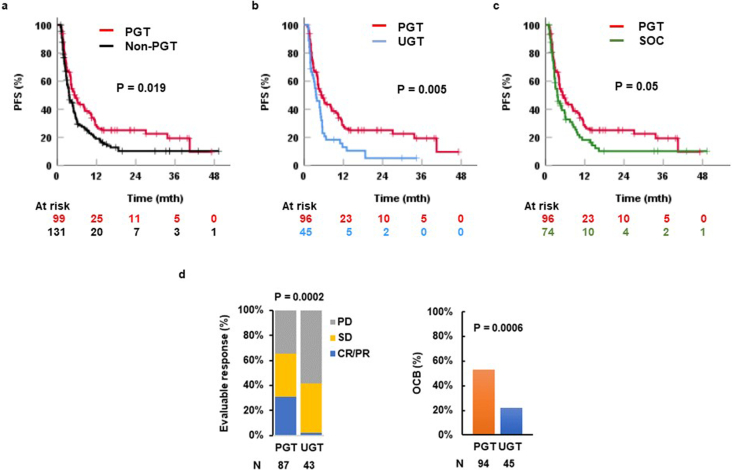
Outcome of treatment with pilocytic astrocytoma excluded from analysis. **a–c**, Progression-free survival (PFS) of PGT comparing with (a) non-PGT, (b) UGT (molecularly unguided therapy) and (c) standard of care (SOC) treatment. **d**, Evaluable response and objective clinical benefit (OCB) of PGT and UGT. Log rank test is used to compare the Kaplan Meier survival curves and Chi-square test is used to compare evaluable response and OCB.

Three patients with pilocytic astrocytomas with atypical aggressive clinical courses and multiple previous disease progressions were treated with PGT. Targetable molecular findings included two with protein tyrosine phosphatase non-receptor type 11 (*PTPN11)* and *FGFR1* mutation and a phosphotyrosine interaction domain-containing protein 1-*BRAF* fusion (Supplementary Data 5). Because pilocytic astrocytoma could have prolonged PFS compared to other tumor types in the cohort, we repeated the analysis excluding these three cases. We found that the improvement in PFS, OR and OCB remained statistically significant when comparing PGT with all other treatments (Extended Data Fig. 3).

### Factors predicting response to PGT

**Fig. 4:**
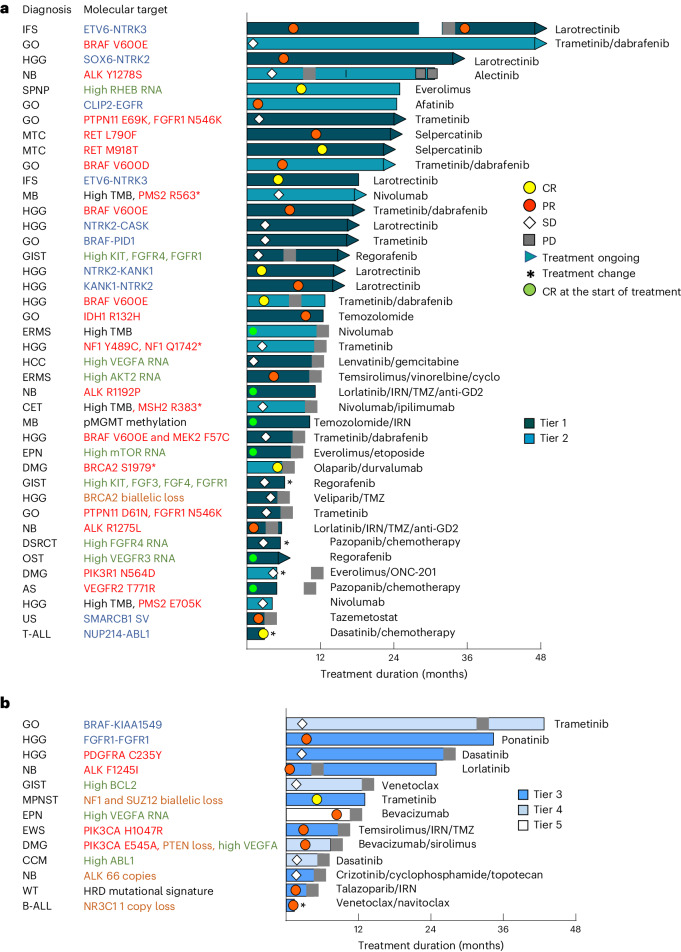
PGT leads to OCB. **a**,**b**, Swimmer plot of 53 PGTs (51 patients) leading to OCB. OCB includes CR, PR and SD for a duration of 24 weeks or longer and ongoing CR of 24 weeks or longer for patients who were in CR at the start of treatment. Forty-one PGTs with tier 1 and 2 recommendations are show in **a** and 12 with tier 3–5 recommendations are shown in **b**. The color of the bars indicates the tier of a PGT recommendation. The symbols indicate the responses and treatment status. The diagnosis and molecular targets for each patient are shown. The types of molecular aberration are denoted by different colored text. The fusion or structural variant (SV) is shown in blue, SNVs in red, high RNA expression in green, copy number variant in brown and other alterations in black. AS, angiosarcoma; B-ALL, B cell acute lymphoblastic leukemia; CCM, clear cell meningioma; CET, CNS embryonal tumor not otherwise specified; DMG, diffuse midline glioma H3K27M-altered; DSRCT, desmoplastic small round cell tumor; ERMS, embryonal rhabdomyosarcoma; GIST, gastrointestinal stromal tumor; GO, glioma other; HCC, hepatocellular carcinoma; HGG, high-grade glioma; IFS, infantile fibrosarcoma; MPNST, malignant peripheral nerve sheath tumor; MTV, medullary thyroid carcinoma; NB, neuroblastoma; SPNP, solid pseudopapillary neoplasm of the pancreas; T-ALL, T cell acute lymphoblastic leukemia; US, undifferentiated sarcoma; WT, Wilms tumor.

**Fig. 5:**
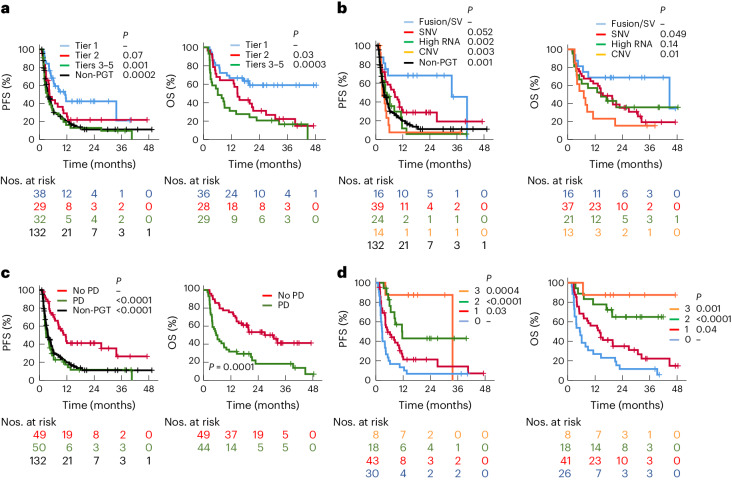
Factors influencing the clinical outcome of PGT. **a**–**d**, PFS and OS stratified according to the tier of PGT (**a**), the types of molecular aberration (**b**), PD from enrollment to the start of PGT (**c**) and the number of favorable prognostic factors (**d**). A two-sided log-rank test was used to compare the Kaplan–Meier survival curves of two groups; the reference subgroup is indicated by a dash.

**Extended Data Fig. 4:**
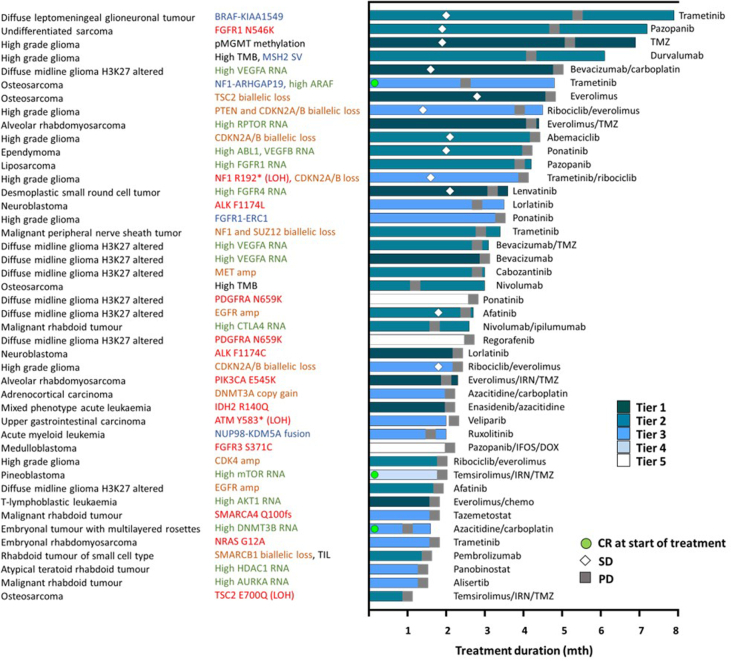
Swimmer plot of 44 precision-guided treatments (PGTs) which did not lead to objective clinical benefit (OCB). Lack of OCB includes patients with disease at the start of treatment who had stable disease (SD) for <24 weeks duration or progressive disease (PD) and patients in complete remission (CR) at the start of treatment with relapse-free duration <24 weeks. The color of the bars indicates tier of a PGT recommendation. Symbols indicate responses and treatment status. The diagnosis and molecular targets for each patient are shown. The types of molecular aberration are denoted by different color text. Fusion or structural variant (SV) is shown in blue, single nucleotide variants in red, high RNA expression in green, copy number variant in brown and other alterations in black.

**Extended Data Fig. 5:**
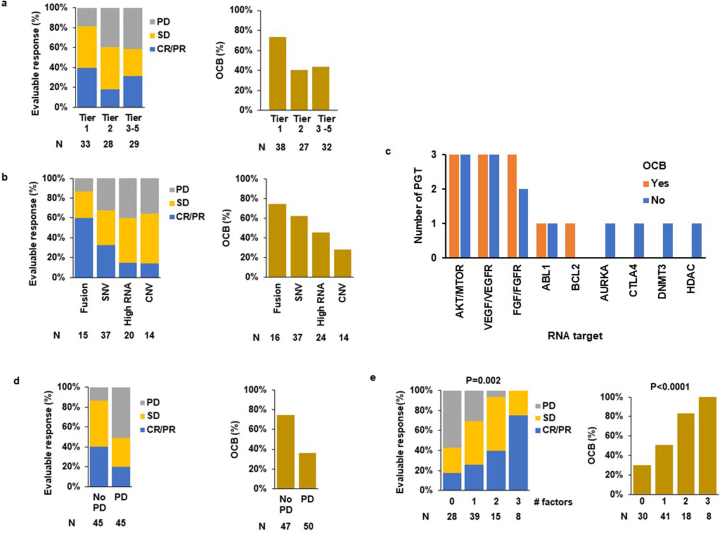
Factors influencing response to precision-guided therapy (PGT). **a**, **b**, Evaluable response and objective clinical benefit (OCB) rate by (a) tier of PGT recommendation and (b) stratified by types of molecular aberration. **c**, The number of PGTs with and without OCB for each specific RNA targets or pathways. **d**, **e**, Evaluable response and OCB rate by (d) progressive disease (PD) from enrolment to the start of PGT and (e) number of favorable prognostic factors. Chi-square test is used to compare evaluable response and OCB rate between 2 groups and the actual P values are presented in Table 1. The P value shown in panel (d) is for Chi-square test for trend. CNV, copy number variation; CR, complete response; PR, partial response; PD, progressive disease; SD, stable disease; SNV, single nucleotide variant; SV, structural variant.

To better understand the characteristics of patients who received a PGT, we examined the target genes, category of molecular aberrations, strength of evidence, tumor type and type of response for each individual patient. We observed responses or prolonged SD across all different scenarios (Fig. 4a,b). Details of CNS tumors with OCB to PGT are provided in Supplementary Table 3. Patients for whom PGT did not lead to OCB are shown in Extended Data Fig. 4. We found that every tier was associated with clinical responses, with response rates highest for tier 1 (39%), but not significantly different to tier 2 (18%; *P* = 0.07) or tiers 3–5 (31%; *P* = 0.49) (Table 1 and Extended Data Fig. 5a). Only one of three patients benefited from tier 5 PGT: a patient with ependymoma (EPN) with high vascular endothelial growth factor A (*VEGFA*) RNA expression with PR to bevacizumab. The remaining two patients progressed rapidly—an H3K27M mutant diffuse midline glioma with *PDGFRA* mutation treated with ponatinib and then regorafenib, and an MB with *FGFR3* mutation given pazopanib, ifosfamide and doxorubicin. Similarly, OCB was also highest for tier 1 (74%) compared with either tier 2 (41%; *P* = 0.008) or tier 3–5 (44%; *P* = 0.01) (Extended Data Fig. 5a). These results translated to improved survival, with tier 1 PGT resulting in longer PFS (tier 1 versus 2; *P* = 0.07 and tier 1 versus tiers 3–5; *P* = 0.001), and OS (tier 1 versus 2; *P* = 0.03 and tier 1 versus tiers 3–5; *P* = 0.0003) when compared to other tiers (Fig. 5a and Table 1), and longer PFS compared to non-PGT (*P* = 0.0002).

**Table 1:** Clinical outcome for patients receiving PGT

	2-year PFS^a^				2-year OS^b^				Evaluable response^c^			OCB		
	*n*	%	95% CI	*P*	*n*	%	95% CI	*P*	*n*	%	*P*	*n*	%	*P*
All	99	27	18–36	—	93	37	27–48	–	90	30	–	97	55	–
**Tier**														
1	38	42	25–59	ref.	36	59	42–76	ref	33	39	ref	38	74	ref.
2	29	22	6–37	0.066	28	31	14–49	0.034	28	18	0.07	27	41	0.008
3–5	32	13	1–25	0.001	29	21	6–35	0.0003	29	31	0.49	32	44	0.01
**Molecular alteration**														
Fusion/SV	16	68	45–91	ref.	16	69	46–92	ref.	15	60	ref.	16	75	ref
SNV	39	29	14–43	0.052	37	35	19–51	0.049	37	32	0.07	37	62	0.36
High RNA	24	5.9	0–17	0.002	21	36	14–57	0.14	20	15	0.006	24	46	0.07
CNV	14	7.7	0–22	0.003	13	15	0–35	0.009	14	14	0.01	14	29	0.01
**Disease status at the start of treatment**														
No PD	49	41	27–56	–	49	53	39–68	–	45	40	–	47	74	–
PD	50	12	2–22	0.00002	44	29	18–41	0.0001	45	20	0.04	50	36	0.0001
**Treatment type**														
Targeted monotherapy/dual therapy	75	32	21–43	–	70	42	30–55	–	72	29	–	73	55	–
Targeted agent + chemotherapy	21	0	–	0.03	20	15	0–33	0.048	16	31	0.87	21	52	0.84
**Cancer type**														
DMG/HGG	31	27	11–43	ref.	28	28	10–46	ref.	30	27	ref.	28	43	ref.
Other CNS	20	44	22–66	0.14	20	65	44–86	0.018	17	24	0.81	21	67	0.10
Solid	43	18	6–31	0.57	40	32	16–47	0.80	38	34	0.50	43	56	0.29
Hematological	5	0	–	0.02	5	20	0–55	0.27	5	40	0.54	5	40	0.84
**Number of favorable factors**^d^														
0	30	6.7	0–16	ref.	26	12	0–24	ref.	28	18	ref.	30	30	ref.
1	43	21	8–35	0.03	41	35	20–50	0.04	39	26	0.45	41	51	0.07
2	18	43	18–68	0.0001	18	65	42–88	0.00002	15	40	0.11	18	83	0.0003
3	8	88	65–100	0.0004	8	88	65–100	0.001	8	75	0.002	8	100	0.0004

^a^PFS is treatment-based and calculated from the start of a PGT to the first disease progression or death.

^b^OS is patient-based and calculated from the start of a PGT to death. For patients who have received more than 1 PGT, the start time is the start of the first PGT.

^c^Evaluable response includes CR and PR for measurable disease and CR for evaluable but nonmeasurable disease.

^d^Favorable factors included tier 1 recommendations, fusion and no PD since enrollment.

*n* refers to the number of PGT analyzed in that group for PFS, evaluable response and OCB analysis. For the OS analysis, *n* refers to the number of patients.

For the analysis involving more than two subgroups, each subgroup was compared with the reference group (ref.) and the *P* value refers to the comparison between the reference group and the specific group. A two-sided log-rank test was used to compare PFS and OS and a two-sided chi-squared test was used to compare proportions for evaluable response and OCB.

We next assessed response and survival based on the PGT target. Patients whose treatment targeted a fusion or SV had the highest response rate (CR/PR) of 60%, compared with single-nucleotide variants (SNVs) (32%; *P* = 0.07), high RNA target expression only (15%; *P* = 0.006) and copy number variation (CNV) (14%; *P* = 0.01) (Extended Data Fig. 5b and Table 1). A similar trend was observed for the OCB. These results correlated with survival outcomes. The 2-year PFS was superior for PGT targeting a fusion/SV (68%), compared with SNV (30%; *P* = 0.057), high RNA expression alone (5.9%; *P* = 0.002) or CNV (7.7%; *P* = 0.003) (Fig. 5b and Table 1). Evaluation of OS demonstrated a similar trend with PGT targeting a fusion/SV leading to a 2-year OS of 69%. PGT targeting a fusion/SV and SNV also led to improved PFS when compared with non-PGT (*P* = 0.001 and *P* = 0.02, respectively).

Of note, 46% (11 of 24) of PGTs targeting high RNA expression alone (not associated with SV, SNV or CNV) led to OCB (Extended Data Table 3), including three ORs. This included a solid pseudopapillary neoplasm of pancreas (everolimus for high Ras homolog enriched in brain (*RHEB*)), EPN (bevacizumab for high *VEGFA*) and rhabdomyosarcoma (temsirolimus/vinorelbine/cyclophosphamide for high *AKT2*). Genes within the AKT/mTOR, VEGF/VEGFR and FGF/FGFR pathways were most frequently targeted for high RNA expression (Extended Data Table 3 and Fig. 5c), with a similar OCB rate (50–60%).

**Extended Data Fig. 6:**
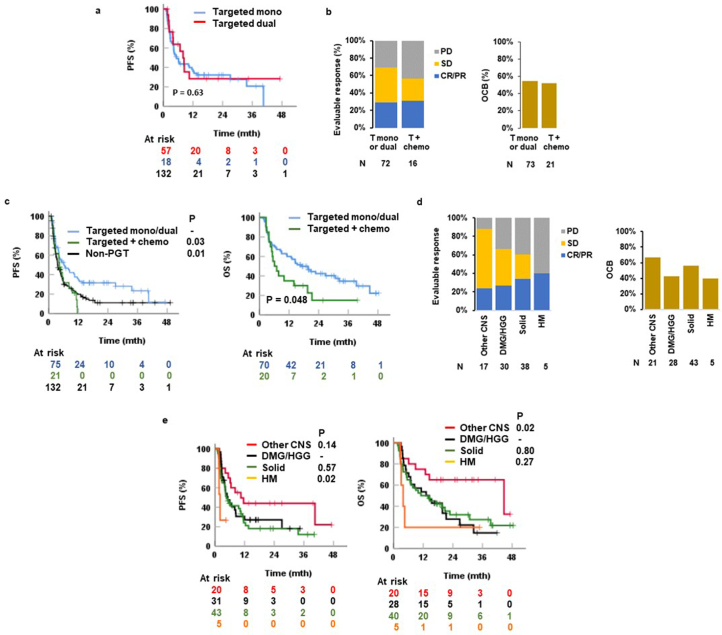
Clinical outcome of precision-guided targeted therapy by treatment and cancer types. **a**, Progression-free survival (PFS) for targeted monotherapy (mono) and targeted dual therapy. **b**, Evaluable response and objective clinical benefit (OCB) rate by type of targeted therapy. **c**, PFS and OS by types of treatment. **d**, Evaluable response and OCB by cancer types. **e**, PFS and OS by cancer types. Two-sided log rank test is used to compare the Kaplan Meier survival curves of 2 groups, with the reference subgroup indicated by a dash and P value next to the group for comparison. Two-sided chi-square test is used to compare evaluable response and OCB rate between 2 groups and the P values are presented in Table 1. CR, complete response; DMG, diffuse midline glioma H3K27M-altered; HGG, high grade glioma; HM, hematologic malignancy; PR, partial response; PD, progressive disease; SD, stable disease.

Of the 99 PGTs administered, 57 were administered as targeted monotherapy, 18 as dual targeted therapy, 21 as combination targeted and chemotherapy and three as chemotherapy. There was no difference in 2-year PFS between targeted monotherapy and dual therapy (32% versus 31%; *P* = 0.6) (Extended Data Fig. 6a). Similar response rates (31% versus 29%; *P* = 0.75) and OCB rates (52% versus 55%; *P* = 0.84) were observed for targeted agents administered in combination with chemotherapy and targeted monotherapy and dual therapy (Extended Data Fig. 6b); however, targeted chemotherapy was associated with significantly inferior survival when compared with targeted monotherapy and dual therapy (2-year PFS 0% versus 32%; *P* = 0.03 and 2-year OS 15% versus 42%; *P* = 0.048) (Table 1 and Extended Data Fig. 6c).

To evaluate the optimal time to initiate PGT, we assessed patients’ disease status at the start of treatment and found a significant correlation with clinical outcome. From enrollment, patients receiving PGT before relapse or progression had a significantly higher response (40% versus 20%; *P* = 0.04) and OCB rates (74% versus 36%; *P* = 0.0001) (Extended Data Fig. 5d). This translated to better survival compared to patients receiving PGT after subsequent disease progression (2-year PFS 42% versus 12%; *P* < 0.0001 and 2-year OS 53% versus 29%; *P* = 0.0002) (Fig. 5c and Table 1) and patients receiving non-PGT (2-year PFS 12%; *P* < 0.0001).

There was no significant difference in outcome between tumor types treated with PGT except for a significantly poorer outcome for patients with HM (*P* = 0.02) (Extended Data Fig. 6d,e). As expected, the uptake of PGT in HM was low (22%), and PGT was given to heavily pretreated patients because of the availability of effective previous salvage therapies. Of the eight patients with HM who received a PGT, three were not evaluable as they were treated for fewer than 4 weeks. Three evaluable patients progressed rapidly and two patients (*NUP214-ABL1* fusion and *NR3C1* monoallelic loss) responded (to dasatinib and venetoclax/navitoclax, respectively) and proceeded to transplant.

Finally, we conducted a multivariate analysis of prognostic factors. Tier 1 evidence, fusion/SV, PGT given before relapse or disease progression, and non-hematological malignancy had independent prognostic significance for PFS, with hazard ratios of 0.43, 0.42, 0.50 and 0.21, respectively (Extended Data Table 4). We next asked whether combinations of independent favorable factors impacted the outcomes for PGT. We identified whether each patient had 0, 1, 2 or 3 of each of the following: tier 1 evidence; fusion/SV; and PGT given before PD. HMs were excluded because of very small numbers. We found that the number of favorable factors was significantly associated with improved response rate, OCB, PFS and OS (Fig. 5d, Extended Data Fig. 5e and Table 1). Patients with three favorable factors demonstrated the highest response rates and OCB of 75% and 100%, respectively. The 2-year PFS was 6.7% for patients with no favorable factors, 21% for patients with one factor (*P* = 0.03), 43% for those with two factors (*P* < 0.0001) and 88% for those with three favorable factors (*P* = 0.0004). The same trend was observed for OS.

## Discussion

It is unclear which children with high-risk cancer benefit from PGT. This study reports a comprehensive outcome analysis of PGT in one of the largest patient cohorts evaluating both response rates and survival outcomes with extended follow-up on a precision medicine trial. Most pediatric precision medicine studies have reported on actionable alterations and patients receiving targeted therapies without objective outcome measurements^5,7–9,14,15^. Three studies (ZERO, GAINS and MAPPYACTS) have reported objective imaging responses^1,3,4^ and one study (INFORM) reported survival outcomes^2^. The lack of comprehensive outcome reporting is a major limitation of pediatric precision medicine studies as discussed in a recent commentary by leaders in the field^16^. In this article, we report both response rates and long-term survival rates, comparing PGT versus non-PGT, showing that response to PGT translates into improved survival; that targeting fusions leads to improved outcomes over other drivers; that early therapy should be considered, particularly for high-priority targets; and that new agents selected based on genomic driver lead to improved outcomes. The clinical uptake for those who received a PGT recommendation (43%) is the highest among pediatric precision oncology studies^2–5,8,14,15^, with some enrolling relapsed and refractory tumors only^4,5,8,14^; others, like ours, include high-risk cancers at diagnosis^2,3,15^. In this study, we have been able to delineate the clinical benefit of PGT more precisely in subgroups of children with high-risk cancer with important implications for treatment.

Our results demonstrate that patients derived clinical benefit from PGT, supported by multiple complementary assessments of clinical outcome, including validating the PFS ratio for the first time as an outcome measure in pediatric precision medicine. We found a significant improvement in PFS for PGT versus non-PGT, UGT or SOC treatment. The INFORM study only observed a difference in PFS between matched targeted treatment and other treatments for those with the highest level of clinical evidence^2^. This could relate to differences between the two studies. In the INFORM study, 9.6% of patients were enrolled at the time of initial diagnosis compared with 42% in this study. INFORM’s approach is to identify therapeutic targets but refrain from making recommendations. In this trial, treatments were recommended after extensive MTB discussion with participation of the treating clinician, potentially contributing to the high uptake. The weekly MTBs have 50–60 attendees, including scientists, clinicians and subject matter experts, with a 10–15 min discussion per patient. We adopted a relatively conservative approach to treatment recommendation with a rigorous literature review and considered recommendations only if there was a reasonable likelihood of drug access. It is possible that these differing approaches impacted treatment decisions and patient responses.

Our comparison of PGT with UGT found a significantly inferior outcome for UGT, which emphasizes the critical role of molecular analysis in guiding therapeutic decision-making. Importantly, 57% of patients treated with an unmatched new therapy had an alternative PGT recommendation. This may be because of easier drug access, or physician or patient preference. Irrespective of the reason for opting for a UGT, our data show clear benefit from PGT, with a striking improvement in response rates (30% versus 2.3%) and 2-year PFS (26% versus 5.2%). This is consistent with the adult literature^11,17–19^. These observations highlight the importance of identifying biomarkers and support the integration of precision medicine into SOC for pediatric patients. This also strongly suggests that clinical trials of new targeted therapies should be biomarker-driven, where possible.

While we showed that PGT significantly improves PFS compared to other treatments, its impact on OS is less clear. The difference in 2-year OS of 38% in PGT did not reach statistical significance when compared with the 2-year OS of non-PGT (24%), UGT (20%) and SOC (23%). This may have been impacted by patients receiving multiple lines of therapy and different salvage therapies; longer follow-up may be required to further assess the impact on OS. In addition, other endpoints, including patient-reported outcomes^20^, quality of life and toxicity measures, should be explored to evaluate the clinical benefits of PGT. More research needs to be undertaken to understand clinician’s decisions not to act on molecular results. There were no obvious differences in diagnoses or targets between the group who received PGT and the group who did not in this study.

Our results help define which treatment strategies provide the greatest clinical benefit for patients. We found that high-level clinical evidence, fusion, non-hematological malignancy and PGT given before relapse or progression, were independent favorable prognostic factors. Importantly, many patients without any of these prognostic factors also derived clinical benefit. However, the differing impact may help clinicians prioritize treatment strategies, especially if multiple targets are identified. Moreover, an 88% 2-year PFS for the eight patients with three prognostic factors (tier 1, fusion and early therapy) are remarkable. This suggests that patients with targetable fusions or treatments supported by high-level clinical evidence should be treated with PGT early, ideally before disease progression. Children with very high-risk cancers should be considered for upfront PGT as part of anticancer treatment at diagnosis.

Limitations to our study include the nonrandomized design, potential influence of clinician bias, challenges with drug access and relatively small numbers of patients for the subgroup analyses. While a randomized trial could reduce some bias, it is not clear that this would ever be feasible in this patient population. Large international collaborations may add further power to allow analyses of smaller subgroups. Lack of availability of drugs and access to appropriate clinical trials also pose a major barrier. Testing these agents through companion basket trials will help with drug access and assessing activity in uniform patient populations. While most patients receiving PGT achieved clinical benefit, most ultimately had disease progression, indicating that further improvements are needed. Future studies could test combination therapies and optimize the timing of PGT. Further effort is required to improve outcomes for children with no targetable findings, or only low-evidence targets. We identified genomic drivers in more than 90% of cases; pharmaceutical companies should be encouraged to develop new therapies that specifically target pediatric tumor drivers.

In conclusion, this study demonstrates that children with high-risk cancers benefit from PGT identified by comprehensive molecular profiling. Treatment with PGT led to improved antitumor activity and survival outcomes, compared to UGT and standard cytotoxic therapies. Treatment strategies should focus on the identification of drivers and early treatment of patients with highly targetable molecular drivers.

## Methods

### Study design and objectives

The PRISM trial (ClinicalTrials.gov registration: NCT03336931) was a multicenter prospective observational cohort study conducted by the Australian ZERO Childhood Cancer Precision Medicine Program and was opened from September 2017. Patients were recruited between September 2017 and December 2020, with data collected prospectively between September 2017 and June 2022. All clinical data were collected by designated clinical research associates and clinicians based at each of the eight pediatric oncology centers in Australia (Sydney Children’s Hospital, The Children’s Hospital at Westmead, John Hunter Children’s Hospital, Queensland Children’s Hospital, Royal Children’s Hospital, Monash Children’s Hospital, Adelaide Women’s & Children’s Hospital and Perth Children’s Hospital) participated in the study. The primary objective was to determine the proportion of patients for whom PGT could be recommended to the treating physician using a comprehensive precision medicine platform within a clinically relevant time frame. Secondary and tertiary objectives included evaluating the treatment response in patients who had received a PGT and the difference in survival between patients receiving PGT and non-PGT.

### Study oversight

The study was conducted in accordance with Good Clinical Practice guidelines and the Declaration of Helsinki and approved by the Hunter New England Human Research Ethics Committee of the Hunter New England Local Health District in Australia (reference no. 2019/ETH00701). Written informed consent for all patients in this study were provided either by the parent or legal guardian for patients younger than 18 years or by patients older than 18 years. There was no participant compensation.

### Patients and tumor samples

Patients younger than 21 years with suspected or confirmed diagnosis of a high-risk malignancy at diagnosis or relapse or refractory, defined as an estimated probability of cure lower than 30%, could be consented and registered on the study. Patients older than 22 years with high-risk pediatric-type cancers could also be registered on approval from the study chair. Patients of any gender or sex were eligible. The sex of a patient was either reported by the guardian or parent or self-reported. No gender information was collected. Sex was not considered in the study design. After trial registration, patient samples were delivered to the central laboratory at the Children’s Cancer Institute (CCI) (Sydney) for processing. A patient was deemed eligible for enrollment when all criteria were satisfied: confirmed high-risk cancer; both tumor and germline sample received at CCI; and sufficient DNA could be extracted for sequencing. High-risk cancer (estimated cure rate of less than 30% based on the published literature) was confirmed by central review of clinical history, histopathology and imaging. Tumor tissue was fresh, snap-frozen or cryopreserved on receipt. A formalin-fixed paraffin-embedded (FFPE) tumor sample was accepted with previous approval from the study chair if this was the only sample available. In those patients who had undergone an allogeneic stem cell transplant (SCT), both the patient germline (usually a skin punch biopsy or a remission sample from a previous clinical time point before SCT) and the surrogate donor germline sample (usually remission blood or bone marrow from the patient after SCT) were sequenced specifically to distinguish tumor-derived somatic variants from patient and donor germline variants. Clinical and demographic data at registration and follow-up were entered into the Labmatrix by Biofortis v.R7 3.2.0 laboratory information management system.

### Molecular profiling

WGS (paired tumor-germline) was conducted for all patients, except when there was insufficient tumor DNA for WGS or only an FFPE tumor sample was available. Targeted panel DNA sequencing was performed for these patients. WTS was conducted in non-FFPE tumor samples whenever RNA of adequate quantity and quality was available. DNA methylation analysis was performed in tumor samples from CNS tumors and appropriate solid tumors. The analytical pipelines for molecular profiling and variant curation for WGS, WTS and methylation^1^, and targeted panel sequencing^21^, have been described previously.

### MTB and PGT recommendations

Patients with molecular alterations that were potentially targetable or could lead to a change or refinement of diagnosis, and patients with reportable germline variants, were presented in the national MTB meeting held fortnightly. The MTB meetings were attended by oncologists, pathologists, clinical geneticists, genetic counselors, basic scientists, bioinformaticians and study managers. The treating oncologist for the patient being discussed was invited to attend and provide a clinical update to facilitate MTB discussion.

Cases for MTB presentation were prepared jointly by the clinical team (consisting of two molecular oncologists and oncology fellows) and the curation scientist team. The clinical team researched and reviewed evidence to support therapeutic options and presented reportable molecular findings and therapeutic options at the MTB. A five-tier system was used to assign the strength of PGT recommendations. Tier 1 referred to evidence from clinical studies of the same cancer type and tier 2 from clinical studies of different cancer types. Patients from these clinical studies can be molecularly selected or unselected. The evidence from tiers 3 and 4 was based on preclinical evidence in the same and different cancer type, respectively. Preclinical models in these studies can be molecularly selected or unselected. Tier 5 was based on the consensus opinion of the MTB. Furthermore, a PGT would only be recommended if there was a possibility of drug access within Australia and pediatric dosing or safety data were available for patients younger than 12 years, for example, at least from a pediatric phase I trial. A weight-adjusted adult dose is acceptable for patients older than 12 years. The final MTB report was generated after the meeting. The treating oncologist made the final treatment decision, in consultation with the family and patient, including consideration for treatments other than the MTB recommendations.

### Treatment and outcome data analysis

Patients who died between registration and MTB presentation were excluded from the outcome analyses. All treatments (including SOC and experimental treatments) and response to treatment were recorded prospectively in Labmatrix. Receipt of a PGT was defined as the patient having received at least one dose of a drug in the same therapeutic class as the PGT recommendation. Drug access route and the treating oncologist’s opinion of clinical benefit of a PGT were also recorded. Data cutoff was set at 30 June 2022.

A treatment was included for the treatment outcome analysis when all three criteria were met: treatment duration lasting 4 weeks or longer; no disease progression within the first 4 weeks of treatment; and treatment response evaluation being available. Treatment response was evaluated using the revised RECIST (v.1.1)^22^ or PERCIST^23^ for solid tumors if positron emission tomography (PET) was the only available imaging study, RANO criteria^24^ for CNS tumors and National Comprehensive Cancer Network guidelines for acute leukemia. To meet the criteria for SD, measurements must have met the SD criteria at a minimum interval of 6 weeks after commencing a treatment. Treatment response evaluation was conducted by central review of imaging reports and, when required, the images.

Measurable diseases were evaluated for CR, PR, SD and PD as defined by the RECIST^22^, RANO^24^ and PERCIST criteria^23^. Measurable disease refers to lesions 10 mm or larger by computed tomography or magnetic resonance imaging and can be accurately measured. Evaluable but non-measurable disease refers to lesions smaller than 10 mm or other sites of disease that cannot be accurately measured, for example, leptomeningeal disease, ascites, pleural or pericardial effusion. The OR for measurable disease was defined as CR or PR as the best response. Evaluable but non-measurable diseases were evaluated for CR, non-CR and non-PD, and PD. CR was defined as disappearance of all non-measurable disease, PD as unequivocal progression of nonmeasurable disease or appearance of new lesion(s) and non-CR/non-PD as persistence of nonmeasurable disease that did not qualify for CR or PD. PR could not be determined for nonmeasurable disease. For the purpose of evaluable response assessment, which included both measurable and non-measurable diseases, SD in measurable disease, and non-CR and non-PD in non-measurable disease, were grouped together as SD. Leukemia response was determined according to blast percentage. CR was defined as less than 5% blasts in bone marrow with no circulating blasts or extramedullary disease, PR as 5% or more and 25% or less blasts or more than 50% relative reduction (with a minimum of 10% absolute reduction) from baseline bone marrow blast count in bone marrow with no circulating blasts or extramedullary disease, and SD as failure to qualify for CR, PR or PD. PD was defined as more than a 25% increase in absolute circulating blast numbers, more than a 25% increase in bone marrow blasts after achieving PR or more than a 5% increase in bone marrow blasts after achieving CR. OCB was evaluated in both measurable and non-measurable disease, and in patients who were in CR at the start of a treatment. OCB was defined as (1) CR, PR or SD lasting for 24 weeks or longer in measurable disease or (2) CR or non-CR and non-PD lasting 24 weeks or longer in non-measurable disease or (3) disease-free duration lasting 24 weeks or longer in patients who were in CR at the start of a treatment. Patients whose best response was SD but stopped or changed treatment before 24 weeks while remaining in SD could not be assessed for OCB and were excluded from the OCB analysis.

Each eligible treatment was evaluated for PFS, defined as the time from the start of that specific treatment to disease progression or recurrence for that treatment, or death from any cause, whichever occurred first. PGTs were analyzed for PFS ratio, which compared PFS achieved by a PGT (PFS2) to the PFS of the most recent previous treatment on which the patient had experienced progression (PFS1). The clinical benefit of a PGT was defined as a PFS ratio (PFS2:PFS1) greater than 1.3 and (PFS2–PFS1) lasting 4 weeks or longer, that is, prolongation of PFS by more than 30% and lasting 4 weeks or longer. A PGT was included for the PFS ratio analysis when all criteria were met: (1) no PD within the first 4 weeks of a PGT; (2) if no PD at the data cutoff, the duration of the PGT must be equal or longer than the preceding comparative treatment; (3) the patient had received a treatment before the PGT and experienced PD; (4) the comparative treatment could be PGT or non-PGT but must be given for relapse and refractory disease; and (5) treatment duration lasting 4 weeks or longer for both treatments.

OS for individual patients in the entire cohort was defined as the time from enrollment to death from any cause. For OS comparison between PGT and non-PGT, patients were categorized according to the first treatment initiated after the MTB and was defined as the time from the start of that specific treatment to death from any cause. Therefore, patients who continued on existing treatment after the MTB and never received a new treatment (PGT or non-PGT) after the MTB were not included in the analysis. For OS comparison between PGT and UGT, only patients who had received a PGT or UGT as the first treatment initiated after the MTB were included. For OS comparison between PGT and SOC, only patients who had received a PGT or SOC as the first treatment initiated after the MTB were included. For OS comparison between PGT subgroups, patients who received more than one PGT were categorized according the first PGT and OS was defined as time from the start of the first PGT to death from any cause.

### Statistical methods

The Kaplan–Meier method was used to analyze survival (OS and PFS) while comparisons were performed using the log-rank test. Multivariate survival analysis was performed using Cox proportional hazards regression analysis. Proportions were compared using a chi-squared test. A two-tailed *P* ≤ 0.5 was considered statistically significant. Statistical analyses were performed using SPSS v.26 (IBM Corporation) or PRISM 9 (GraphPad Software).

### Reporting summary

Further information on research design is available in the Nature Portfolio Reporting Summary linked to this article.

## Online content

Any methods, additional references, Nature Portfolio reporting summaries, source data, extended data, supplementary information, acknowledgements, peer review information; details of author contributions and competing interests; and statements of data and code availability are available at 10.1038/s41591-024-03044-0.

### Supplementary information

Supplementary Information Supplementary Tables 1–3.

Reporting Summary

Supplementary Data 1 Supplementary data S1–S5.

## Figures and Tables

**Extended Data Table 1:**
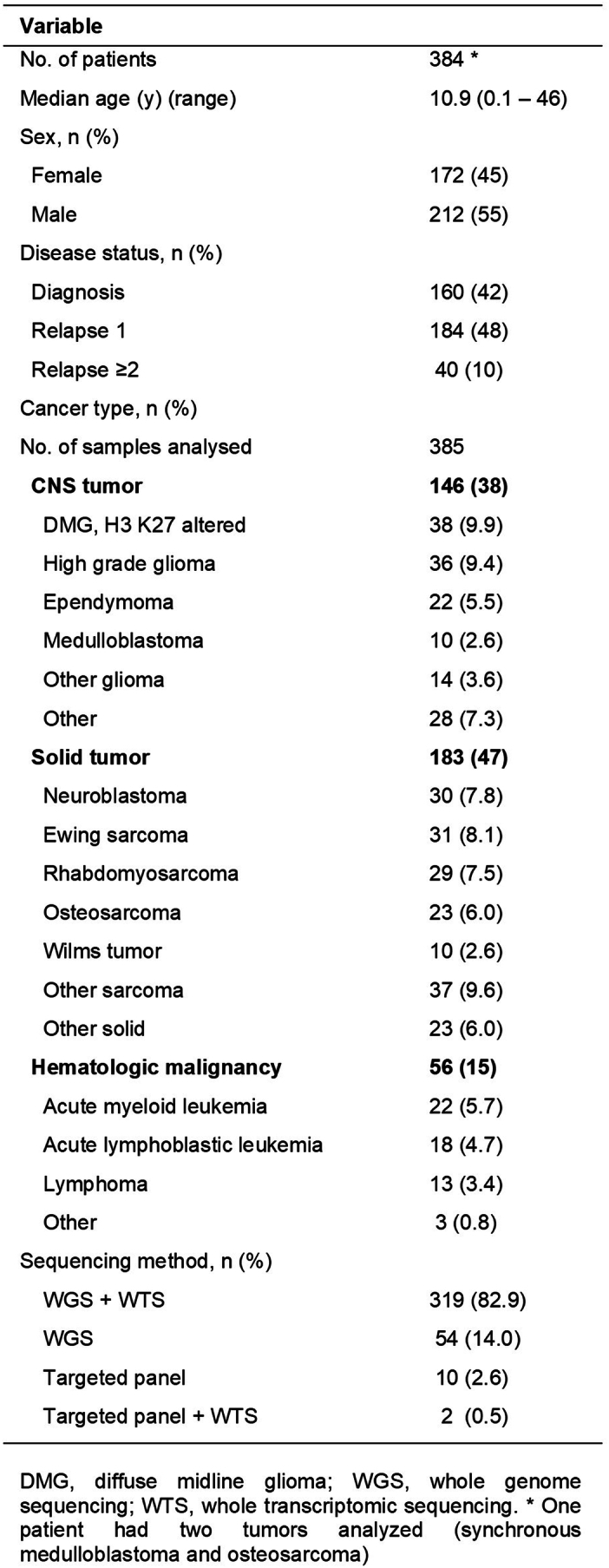
Baseline characteristics and sequencing method

**Extended Data Table 2:**
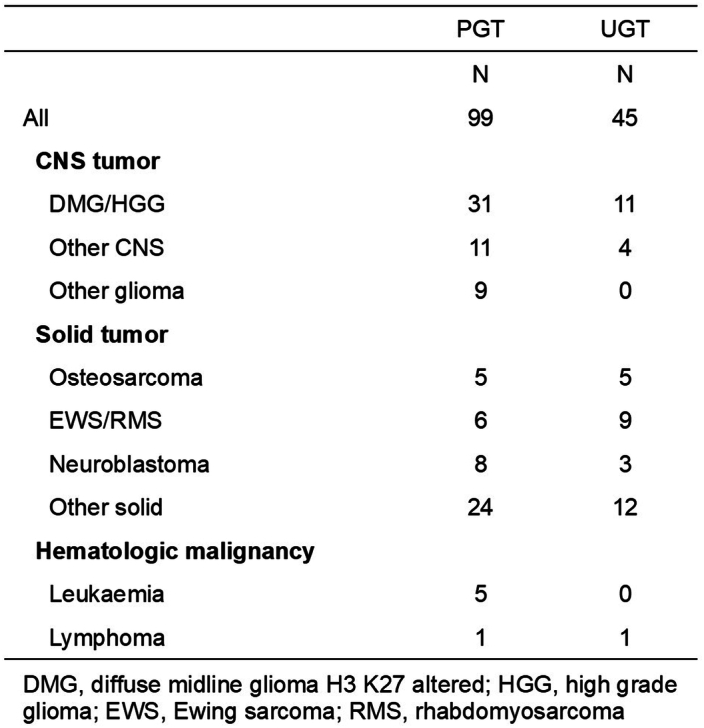
Tumor types within the PGT and UGT groups

**Extended Data Table 3:**
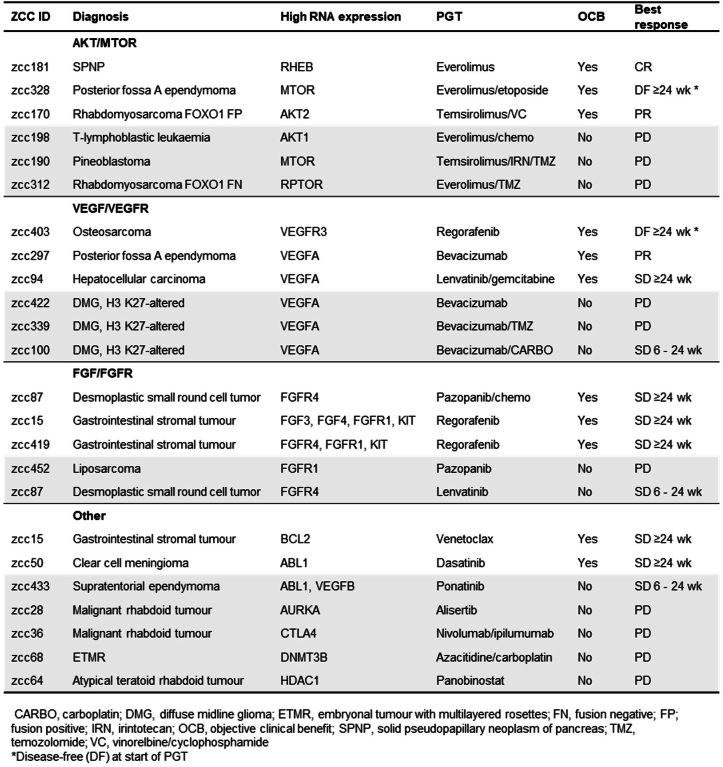
Details and responses of 24 PGTs targeting high RNA expression

**Extended Data Table 4:**
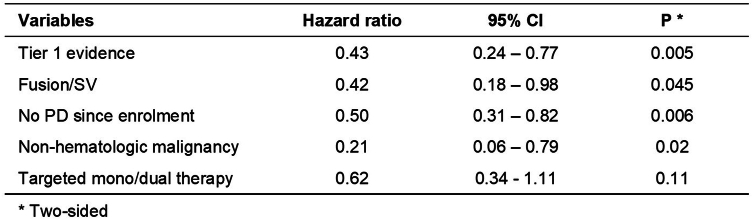
Multivariate Cox regression analysis of factors influencing PFS

## Data Availability

The WGS, RNA-seq and DNA methylation data generated by this study are available from the European Genome-phenome Archive under accession nos. EGAS00001004572 and EGAS00001007029. The Supplementary Data 1 file contains individual patient demographic data (S1), PGT tiers of recommendation (S2), details of PGT (S3) and UGT (S4), details of CNS tumors with PGT benefit (S5) and reportable molecular aberrations detected using panel sequencing (S6).

## Code availability

The code used for the molecular analysis has been previously reported^1^ and is available at https://github.com/CCICB/2020-hrPC-landscape.
