# Supplementary material for: Precision-guided treatment in high-risk pediatric cancers
Source: Nat Med. 2024 Jun 6;30(7):1913–22. doi: 10.1038/s41591-024-03044-0 (PMC11271405; doi:10.1038/s41591-024-03044-0)
Supplement: Supplementary file 1 — Supplementary Tables 1–3. [file 41591_2024_3044_MOESM1_ESM.pdf]

nature medicine

Supplementary information https://doi.org/10.1038/s41591-024-03044-0

Precision-guided treatment in high-risk pediatric cancers

In the format provided by the authors and unedited

Table of Contents

Supplementary table 1.	Availability of clinical tests for PGT targets.
Supplementary table 2.	Details of salvage therapy.
Supplementary table 3.	Details of 40 PGTs received by 38 patients with CNS tumor and clinical benefit.

Supplementary table 1. Availability of clinical tests for PGT targets.

Target	PGT N	Clinical tests Available N	Performed N	Results	Target (test)
Fusion/SV	17	3	3	3 negative	2 ETV6-NTRK3 (ETV6 break-apart FISH)<br>1 CASK-NTRK2 (RNA-seq panel)
SNV	46	7	6	1 equivocal	1 IDH1 (IHC)
				5 positive	1 IDH2 (NGS panel)<br>2 RET (NGS panel)<br>2 BRAF V600E (IHC) *
CNV	19	1	1	1 positive	1 ALK amp. (SNP array)
RNA	27	0	-	-	
Other	8	2	0	-	
Total	117	13	10	6 positive<br>3 negative<br>1 equivocal	

* One additional CNS tumour had both a BRAF V600E and MEK2 mutation, and only the BRAF V600E was identified by clinical testing. The MEK2 mutation suggested that the tumour may be resistant to BRAF inhibitor monotherapy and the patient received dual BRAF/MEK inhibitor therapy as recommended.

Supplementary table 2. Details of salvage therapy.

	PGT		Non-PGT	
	N	%	N	%
Total no. of patients	89		84	
No salvage therapy	62	70%	46	55%
Salvage therapy	27	30%	38	45%
No salvage therapy				
Alive with no PD	23	37%	10	22%
No salvage after PD	38	61%	33	72%
Death from TRM	1	2%	1	2%
Unknown	0	0%	2	4%
Salvage therapy				
By treatment type				
1 line				
PGT	5	19%	4	11%
UGT	5	19%	7	18%
SOC	1	3%	13	34%
Other experimental	6	22%	2	5%
2 lines	6	22%	8	21%
≥3 lines	4	15%	4	11%
By cancer type				
DMG/HGG	10	37%	3	8%
Other CNS	6	22%	7	18%
Solid tumor	11	41%	6	16%
Hematologic	0	0%	22	58%

DMG, diffuse midline glioma, H3K27M mutant; HGG, high grade glioma; PD, progressive disease; PGT, precision-guided treatment; SOC, standard of care; TRM, treatment related mortality; UGT, molecularly unguided therapy.

Note: Salvage therapy refers to treatment given for relapse or disease progression following one line of treatment post MTB discussion.

Supplementary table 3. Details of 40 PGTs received by 38 patients with CNS tumor and clinical benefit.

Histologic diagnosis	Methylation class	Methylation interpretation	PGT	PGT target	PGT best response	PGT OCB
High grade glioma WHO G4	Glioblastoma, IDH wildtype, subclass midline	Match	Veliparib/TMZ	BRCA2 biallelic loss	SD	Yes
High grade glioma WHO G4	Diffuse midline glioma H3 K27M mutant	Low score match	Bevacizumab/sirolimus	PIK3CA E545A, PTEN loss, high VEGFA	PR	Yes
High grade glioma WHO G4	Not performed	NA	Trametinib/dabrafenib	BRAF V600E	CR	Yes
Glioblastoma WHO G4	Glioblastoma, IDH wildtype, H3.3 G34 mutant	Match	Ribociclib/everolimus	PTEN biallelic, loss, CDKN2A/B biallelic loss	SD	No
Glioblastoma WHO G4	(anaplastic) pleomorphic xanthoastrocytoma	No match	Larotrectinib	NTRK2-KANK1	CR	Yes
High grade glioma WHO 3/4	Glioblastoma, IDH wildtype, H3.3 G34 mutant	Match	Dasatinib	PDGFRA C235Y	SD	Yes
High grade glioma WHO 3/4	No methylation class with score >= 0.30	No match	Trametinib/dabrafenib	BRAF V600E	SD	Yes
High grade glioma WHO G3	Glioblastoma, IDH wildtype, subclass RTK III	Low score match	Ribociclib/everolimus	CDKN2A/B biallelic loss	SD	No
High grade glioma WHO G3	Glioblastoma, IDH wildtype, subclass RTK III	Match	Trametinib	NF1 Y489C, NF1 Q1742*	SD	Yes
High grade glioma	Glioblastoma, IDH wildtype, H3.3 G34 mutant	Match	TMZ	pMGMT methylation	SD	No
High grade glioma	LGG, subclass hemispheric pilocytic astrocytoma and ganglioglioma	No match	Ponatinib	FGFR1-FGFR1	PR	Yes
Anaplastic astrocytoma WHO G3	(anaplastic) pleomorphic xanthoastrocytoma	Discordant	Larotrectinib	KANK1-NTRK2	PR	Yes
Anaplastic astrocytoma WHO G3 †	Glioblastoma, IDH wildtype, subclass MYCN	Low score match	Trametinib/ribociclib	NF1 R192* (LOH), CDKN2A/B bialleic loss	SD	No
Anaplastic astrocytoma WHO G3 †	Glioblastoma, IDH wildtype, subclass MYCN	Low score match	Abemaciclib	CDKN2A/B biallelic loss	SD	No
Anaplastic astrocytoma WHO G3	No methylation class with score >= 0.30	No match	Nivolumab	High TMB, PMS2 E705K	SD	NE
PXA WHO G3	No methylation class with score >= 0.30	No match	Trametinib/dabrafenib	BRAF V600E, MEK2 F57C	SD	Yes
Glioneuronal tumour WHO G4	No methylation class with score >= 0.30	No match	Larotrectinib	NTRK2-CASK	SD	Yes
Anaplastic glioneuronal tumor	Diffuse leptomeningeal glioneuronal tumor	Discordant	Larotrectinib	SOX6-NTRK2	PR	Yes
DMG, H3 K27M mutant	Diffuse midline glioma H3 K27M mutant	Match	Everolimus/ONC-201	PIK3R1 N564D	SD	NE
DMG, H3 K27M mutant ††	Diffuse midline glioma H3 K27M mutant	Match	Olaparib/durvalumab	BRCA2 S1979*	CR	Yes
DMG, H3 K27M mutant ††	Diffuse midline glioma H3 K27M mutant	Match	Bevacizumab/carboplatin	High VEGFA RNA	SD	No
DMG, H3 K27M mutant	Diffuse midline glioma H3 K27M mutant	Low score match	Afatinib	EGFR amp	SD	No
DLGNT	Diffuse leptomeningeal glioneuronal tumor, subtype 2	Match	Trametinib	BRAF-KIAA1549	SD	No
Glioneuronal tumour	LGG, subclass hemispheric pilocytic astrocytoma and ganglioglioma	Low score match	Afatinib	CLIP2-EGFR	PR	Yes
Ganglioglioma	No methylation class with score >= 0.30,	No match	Trametinib/dabrafenib	BRAF V600E	SD	Yes
Ganglioglioma	control tissue, reactive tumor microenvironment	Discordant (low purity)	Trametinib/dabrafenib	BRAF V600D	PR	Yes
Diffuse glioma WHO G2/3	Glioblastoma, IDH wildtype, subclass midline	Discordant	Trametinib	BRAF-KIAA1549	SD	Yes
Astrocytoma WHO G2	Not performed	NA	Temozolomide	IDH1 R132H	PR	Yes
Pilocytic astrocytoma	Low grade glioma, subclass posterior fossa pilocytic astrocytoma	Match	Trametinib	BRAF-PID1	SD	Yes
Pilocytic astrocytoma	Low grade glioma, subclass midline pilocytic astrocytoma	Match	Trametinib	PTPN11 E69K, FGFR1 N546K	SD	Yes
Pilocytic astrocytoma	Low grade glioma, subclass midline pilocytic astrocytoma	Match	Trametinib	PTPN11 D61N, FGFR1 N546K	SD	Yes
MB, non WNT/non-SHH	No methylation class with score >= 0.30	No match	IRN/TMZ	pMGMT methylation	NE	Yes
MB, SHH-activated	Medulloblastoma, subclass SHH A (children and adult)	Match	Nivolumab	PMS2 R563*, high TMB	SD	Yes
Posterior fossa A ependymoma	Ependymoma, posterior fossa group A	Match	Everolimus/etoposide	High mTOR RNA	NE	Yes
Posterior fossa A ependymoma	Ependymoma, posterior fossa group A	Match	Bevacizumab	High VEGFA RNA	PR	Yes
Supratentorial ependymoma	Ependymoma, RELA fusion	Match	Ponatinib	High ABL1, VEGFB RNA	SD	No
ETMR	Embryonal tumor with multilayered rosettes	Match	Azacitidine/carboplatin	High DNMT3B RNA	NE	No
Pineoblastoma	Pineoblastoma group B	Match	Temsirolimus/IRN/TMZ	High mTOR RNA	NE	No
CNS embryonal tumour	No methylation class with score >= 0.30	No match	Nivolumab/ipilumumab	High TMB, MSH2 R383*	SD	Yes
Clear cell meningioma	No methylation class with score >= 0.30	No match	Dasatinib	High ABL1 RNA	SD	Yes

Abbreviation: CR, complete response; DLGNT, diffuse leptomeningeal glioneuronal tumor; DMG, diffuse midline glioma; ETMR, embryonal tumor with multilayered rosettes IRN, irinotecan; LGG, low grade glioma; LOH, loss of heterozygosity; MB, medulloblastoma; NE, not evaluable; PR, partial response; PXA, pleomorphic xanthoastrocytoma; SD, stable disease; TMZ, temozolomide; Note: † and ††: same patient receiving different PGT
