# Supplementary material for: Precision-guided treatment in high-risk pediatric cancers
Source: Nat Med. 2024 Jun 6;30(7):1913–22. doi: 10.1038/s41591-024-03044-0 (PMC11271405; doi:10.1038/s41591-024-03044-0)
Supplement: Supplementary file 2 — Reporting Summary [file 41591_2024_3044_MOESM2_ESM.pdf]

nature portfolio

Corresponding author(s): David Ziegler

Last updated by author(s): Apr 26, 2024

Reporting Summary

Nature Portfolio wishes to improve the reproducibility of the work that we publish. This form provides structure for consistency and transparency in reporting. For further information on Nature Portfolio policies, see our Editorial Policies and the Editorial Policy Checklist.

Statistics

For all statistical analyses, confirm that the following items are present in the figure legend, table legend, main text, or Methods section.

n/a	Confirmed	
☐	☒	The exact sample size (*n*) for each experimental group/condition, given as a discrete number and unit of measurement
☒	☐	A statement on whether measurements were taken from distinct samples or whether the same sample was measured repeatedly
☐	☒	The statistical test(s) used AND whether they are one- or two-sided<br>*Only common tests should be described solely by name; describe more complex techniques in the Methods section.*
☒	☐	A description of all covariates tested
☐	☒	A description of any assumptions or corrections, such as tests of normality and adjustment for multiple comparisons
☐	☒	A full description of the statistical parameters including central tendency (e.g. means) or other basic estimates (e.g. regression coefficient) AND variation (e.g. standard deviation) or associated estimates of uncertainty (e.g. confidence intervals)
☐	☒	For null hypothesis testing, the test statistic (e.g. *F*, *t*, *r*) with confidence intervals, effect sizes, degrees of freedom and *P* value noted<br>*Give P values as exact values whenever suitable.*
☒	☐	For Bayesian analysis, information on the choice of priors and Markov chain Monte Carlo settings
☒	☐	For hierarchical and complex designs, identification of the appropriate level for tests and full reporting of outcomes
☒	☐	Estimates of effect sizes (e.g. Cohen's *d*, Pearson's *r*), indicating how they were calculated

Our web collection on statistics for biologists contains articles on many of the points above.

Software and code

Policy information about availability of computer code

Data collection	Clinical data collection was performed within LabMatrix by Biofortis, version R7.3.2.0
Data analysis	Code availability: Software and scripts related to this publication are available at https://github.com/CCICB/2020-hrPC-landscape.<br>Statistical analyses were performed using the IBM SPSS Statistics 26 or GraphPad PRISM 9 software.

For manuscripts utilizing custom algorithms or software that are central to the research but not yet described in published literature, software must be made available to editors and reviewers. We strongly encourage code deposition in a community repository (e.g. GitHub). See the Nature Portfolio guidelines for submitting code & software for further information.

Data

Policy information about availability of data

All manuscripts must include a data availability statement. This statement should provide the following information, where applicable:

- Accession codes, unique identifiers, or web links for publicly available datasets
- A description of any restrictions on data availability
- For clinical datasets or third party data, please ensure that the statement adheres to our policy

WGS, RNAseq and DNA methylation data generated by this study are available from the European Genome-phenome Archive under accession number EGAS00001004572 and EGAS00001007029. A Supplementary Data file containing individual patient demographic data (S1), PGT tiers of recommendation (S2), details of PGT (S3) and UGT (S4), details of CNS tumors with PGT benefit (S5) and reportable molecular aberrations detected by panel sequencing (S6) is included.

Research involving human participants, their data, or biological material

Policy information about studies with human participants or human data. See also policy information about sex, gender (identity/presentation), and sexual orientation and race, ethnicity and racism.

Reporting on sex and gender	This is a prospective observational cohort study where patients of any gender or sex were eligible for enrolment, provided other eligibility criteria for the study were met. Gender and sex were not considered in the study design. Information on sex but not gender was collected for the study. Sex was either reported by a guardian/parent or self-reported. No sex-based analyses have been performed as there was no evidence to suggest that the results of this study would be influenced by sex of the human research participants and the study lacked statistical power to disaggregate each cohort.
Reporting on race, ethnicity, or other socially relevant groupings	This study did not report on race, ethnicity or other socially relevant groups.
Population characteristics	Age <21 years, any gender, diagnosis of a malignancy with an estimated probability of cure less than 30%, at any time of disease course (diagnosis, relapse, progression), any prior treatment with no segregation of treatment categories
Recruitment	This is an observational study where pediatric participants, who were eligible as per study criteria, were identified by the treating clinician. While a small number of patients were excluded from tumor analysis because of lack of suitable tumor material, this is unlikely to impact on the results. It is possible that investigators had a bias to enrol patients whose tumors they considered more likely to harbor targetable lesions. However, there was a broad spectrum of tumor types included, and the number and types of patients enrolled were in keeping with the anticipated national annual incidence of high-risk tumor types.
Ethics oversight	The study was conducted in accordance with Good Clinical Practice guidelines and the Declaration of Helsinki and approved by the Hunter New England Human Research Ethics Committee of the Hunter New England Local Health District in Australia (reference no. 2019/ETH00701). Written informed consent for all patients in this study were provided either by the parent/legal guardian for patients aged <18 years or by patients aged ≥18 years. There was no participant compensation.

Note that full information on the approval of the study protocol must also be provided in the manuscript.

Field-specific reporting

Please select the one below that is the best fit for your research. If you are not sure, read the appropriate sections before making your selection.

☒ Life sciences ☐ Behavioural & social sciences ☐ Ecological, evolutionary & environmental sciences

For a reference copy of the document with all sections, see nature.com/documents/nr-reporting-summary-flat.pdf

Life sciences study design

All studies must disclose on these points even when the disclosure is negative.

Sample size	This is a prospective observational cohort study. Sample size determination was performed for precision-guided therapy recommendation rate. At the time of study design the literature indicated that 90% of the enrolled patients should have adequate tumour tissue for molecular analysis (Harris, JAMA Oncology 2016; Parsons, JAMA Oncology 2026), up to 45% of relapsed/refractory tumour samples would have actionable mutations (Mody, JAMA 2015; Chang, Clinical Cancer Research 2016).<br>We therefore hypothesised that:<br>• 10% of the tumours from enrolled patients cannot be profiled using any of the methods because the quantity and/or quality of the submitted tumour tissue would be inadequate for analysis, i.e., 90% of the patients would have adequate tumour tissue<br>• 50% of the patients with adequate tumour tissue would have a targetable alteration detected by molecular profiling<br>• 60% of the above patients would receive a precision-guided therapy recommendation<br>• 75% of the recommendations would be made within a clinically relevant timeframe<br>Hence the predicted proportion of enrolled patients who would receive a recommendation within a clinically relevant timeframe was 20%. Feasibility is therefore defined as 20% or more of the patients receiving a recommendation for personalised treatment within a clinically relevant timeframe. A sample size of 246 would provide a 95% confidence interval of +/-5% for a 20% recommendation rate.
Data exclusions	The following data is excluded from outcome analyses:<br>1. Patients who died between study registration and molecular tumor board presentation were excluded, as these patients could not be evaluated for the impact of molecular profiling on outcome.<br>2. Any treatment (both precision-guided therapy and other therapies) where treatment duration was less than 4 weeks, as the effectiveness of these treatments could not be evaluated with such short treatment duration<br>3. Any treatment (both precision-guided therapy and other therapies) where disease progression occurred within the first 4 weeks of treatment, as the patients had rapidly progressive disease and the effectiveness of a treatment could not be evaluated appropriately<br>The exclusion criteria were pre-established.
Replication	This is a prospective observational cohort study where limited tumor samples, majority of which from biopsy, underwent whole genome and transcriptomic sequencing, and DNA methylation, and replicating the molecular profiling on individual tumor samples was not feasible and applicable. However, all samples underwent same sequencing, bioinfomatics and analysis pipelines.

Randomization	This is a study where tumor sample of enrolled patients underwent prospective and uniform sequencing and analysis to evaluate feasibility and clinical utility. While patients received precision-guided treatment recommendations, treatment was at the discretion of the clinician. The study design was therefore prospective observational and did not involve randomization.
Blinding	This is a study where tumor sample of enrolled patients underwent prospective sequencing and analysis to evaluate feasibility and clinical benefit. The study design was therefore prospective observational and did not involve blinding.

Reporting for specific materials, systems and methods

We require information from authors about some types of materials, experimental systems and methods used in many studies. Here, indicate whether each material, system or method listed is relevant to your study. If you are not sure if a list item applies to your research, read the appropriate section before selecting a response.

Materials & experimental systems

n/a	Involved in the study	
☒	☐	Antibodies
☒	☐	Eukaryotic cell lines
☒	☐	Palaeontology and archaeology
☒	☐	Animals and other organisms
☐	☒	Clinical data
☒	☐	Dual use research of concern
☒	☐	Plants

Methods

n/a	Involved in the study	
☒	☐	ChIP-seq
☒	☐	Flow cytometry
☒	☐	MRI-based neuroimaging

Clinical data

Policy information about clinical studies

All manuscripts should comply with the ICMJE guidelines for publication of clinical research and a completed CONSORT checklist must be included with all submissions.

Clinical trial registration	NCT03336931
Study protocol	https://clinicaltrials.gov/ct2/show/NCT03336931
Data collection	Patients were recruited between September 2017 and December 2020, with data collected prospectively between September 2017 and June 2022. All clinical data presented was collected by designated clinical research associates and clinicians based at each of the 8 clinical centres where the children with malignancy are managed in Australia; Sydney Children's Hospital (Sydney), Children's Hospital at Westmead (Sydney), Royal Children's Hospital (Melbourne), Monash Hospital for Children (Melbourne), Queensland Children's Hospital (Brisbane), Women's and Children's Hospital (Adelaide), Perth Children's Hospital (Perth), John Hunter Children's Hospital (Newcastle).
Outcomes	The primary, secondary and tertiary clinical outcomes described in the manuscript were predefined in the protocol as below:<br>A. Primary outcome: Proportion of patients receiving a recommendation for personalised therapy within a clinically relevant timeframe<br>B. Secondary outcomes:<br>1. Proportion of tumour samples found to have actionable molecular alterations<br>2. Proportion of patients who subsequently receive the recommended personalised therapy<br>3. Response rate to recommended personalised therapy<br>C. Tertiary outcomes:<br>Difference in outcome between patients receiving recommended personalised therapy and those who do not:<br>1. Difference in progression-free and overall survival between the two groups<br>2. Progression-free interval (PFI) compared with patient's previous PFI for patients who had previous documented PFI prior to commencing recommended personalised therapy, with in an increase in PFI by 30% considered to be effective
